# Rigorous Morphological Studies Confirm That the Classical Object of Pest Control *Chilocorus kuwanae* Is the Same Species as *Ch. renipustulatus* (Coleoptera: Coccinellidae)

**DOI:** 10.3390/insects11060368

**Published:** 2020-06-13

**Authors:** Andrzej O. Bieńkowski, Marina J. Orlova-Bienkowskaja

**Affiliations:** A.N. Severtsov Institute of Ecology and Evolution, Russian Academy of Sciences, Moscow 119071, Russia; bienkowski@yandex.ru

**Keywords:** biocontrol, taxonomy, morphology, beetles, ladybirds, ladybug, *Chilocorus*

## Abstract

The ladybug *Chilocorus kuwanae*, which was described in Japan, has been used for biological control of pests for 100 years. *Chilocorus kuwanae* was recently synonymized with *Ch. renipustulatus* described in Europe. The synonymy was based on the examination of few specimens. Our aim is to verify this synonymy. We studied all characters previously used to distinguish these taxa: eight metric and nine qualitative characters. Examination of 107 specimens from Japan and Sakhalin and 174 specimens from Europe showed that the ranges of variability in all characters in Asian and European specimens strongly overlap. There are no characters with interspecific hiatuses. Analysis with Amadon’s criteria showed that Asian and European specimens also do not represent different subspecies. Conclusions: (1) No differences between the specimens from Asia (Japan and Sakhalin) and Europe were found at specific or subspecific levels. *Chilocorus kuwanae* is a junior synonym of *Ch. renipustulatus*. (2) The releases of “*Chilocorus kuwanae*” in Europe and the Caucasus did not represent classical biological control since the same species was native to these regions. (3) A thorough taxonomical revision with the study of morphological variability should be conducted before the introduction of any species to new regions. (4) Taxonomical conclusions based on morphological studies should be confirmed by statistical methods.

## 1. Introduction

Knowledge of the taxonomy, diagnostic features, and geographical distribution of the species used for biological control of plant pests is of great practical importance. The ladybug *Chilocorus kuwanae* Silvestri, 1909 has been used for the biological control of coccids for more than 100 years [1,2,3,4,5,6,7,8,9,10,11,12,13,14,15,16,17,18,19,20,21,22,23,24,25,26,27,28,29,30,31,32,33]. However, despite this, the taxonomy of this species is still highly problematic, and its geographic distribution is uncertain [34].

Silvestri [7], Kamiya [33], Savoyskaya [35], and Kuznetsov [36,37] noted that *Ch. kuwanae* is morphologically similar to the widely distributed Palearctic species *Ch. renipustulatus* (Scriba, 1791). *Chilocorus kuwanae* was originally described by Silvestri [7] based on uncertain number of specimens received alive from the Japanese entomologist Shinkai Inokichi Kuwana (1872–1933) for biological control of *Pseudaulacaspis pentagona* (Targioni Tozzetti, 1886) in Italy. Therefore, the type specimens were syntypes, and type locality is Japan. It is not known where Coccinellidae specimens from the collection by Silvestri are deposited [38]. We sent a request about the type of *Ch. kuwanae* to the “Museo entomologico Filippo Silvestri”, but this type was not found there. Silvestri [7] did not study any specimens of *Ch. renipustulatus* and mentioned the characteristics of the latter after Ganglbauer [39] and Mulsant [40].

The type locality of *Ch. renipustulatus* was not indicated originally [41]. Most likely, it is in Hessen-Darmstadt (Germany), where the author, Ludwig Gottlieb Scriba (1736–1804), worked. It is unknown where the L.G. Scriba collection was deposited [38]. None of the types of taxa described by Scriba, including *Ch. renipustulatus*, are currently known.

According to Kuznetsov [37] and Kovář [42], the native range of *Ch. kuwanae* includes the Far East (Primorsky Krai), Sakhalin, Kuriles, Japan, Korean Peninsula, and China, while *Ch. renipustulatus* is widely distributed in Europe, Central Asia, the Caucasus, China, Mongolia, Siberia, and the Far East (east to the Amur Region). Therefore, it is generally assumed that the geographical ranges of *Ch. kuwanae* and *Ch. renipustulatus* almost do not overlap. No cases of the establishment of *Ch. kuwanae* in Central and Eastern Europe have been recorded [7,8,9,10,15].

A number of external morphology and male genitalia features were indicated as distinguishing between these two species [33,36,37,43]. However, while working on the key to Coccinellidae for European Russia and the Russian Caucasus, Bieńkowski found that it was impossible to distinguish *Ch. renipustulatus* from *Ch. kuwanae* [34] and formally synonymized them. However, this conclusion was based on the examination of only 10 specimens from Europe and four specimens from Japan without statistical treatment. The aim of the present study is to verify this synonymy by examination of large number of specimens and statistical analysis of the geographic variability in morphology.

## 2. Materials and Methods

### 2.1. Material Examined

We studied adult beetles from the following collections: Zoological Institute of Russian Academy of Sciences, St. Petersburg (ZIN), Zoological Museum of Moscow State University (ZMMU), All-Russian Institute of Plant Quarantine, Moscow region (IPQ), Naturhistorisches Museum Wien (NHMW), Far Eastern branch of Russian Academy of Sciences, Vladivostok (FERAS), Naturkundemuseum Erfurt (NME), All-Russian Institute of Plant Protection, St. Petersburg (IPP), and Siberian Zoological Museum, Novosibirsk (SZM). In addition, specimens were presented to us by H. W. Cho (Republic of Korea) and Sh. Shigehiko (Japan). Materials from the first author’s collection were also studied.

We examined materials from Central and Eastern Europe and from the Far East islands (Japan and Sakhalin) (Table 1) because *Chilocorus renipustulatus* occurs all over Europe and does not occur in Japan and Sakhalin, while *Ch. kuwanae* occurs in Japan and Sakhalin and does not occur in Central and Eastern Europe [37].

We do not designate the neotype of *Ch. renipustulatus* because it is no doubt that all specimens from Central Europe belong to *Ch. renipustulatus* (no other species from this group are recorded from C. Europe). We also do not designate the neotype of *Ch. kuwanae* because it is no doubt that all examined specimens from Japan and Sakhalin belong to the taxon, described as *Ch. kuwanae.*

### 2.2. Methods of Examination of Morphology

All specimens were placed into water with a small amount of detergent for 12 h to soften before preparation and clean the surface of the body (old specimens were often contaminated, which made the puncturation and microsculpture difficult to observe). All specimens were dissected since we did not find any external differences between males and females; penis guides and parameres of all males were prepared.

The structural details of all beetles were studied by one author to reduce the subjectivity of the assessment of characters [44]. Individuals from different regions were studied in random order to reduce the influence of systematic error in the assessment of characters (drift of understanding of characters with continuous variability during processing of the material). The terminology used for the details of external morphology is accepted according to Kamiya [33], with the exception of “lateral parts of pronotum,” which we call “anterior lateral lobes of pronotum”. Details of the structure of male genitalia are accepted according to Li et al. [45].

**Figure 1:**
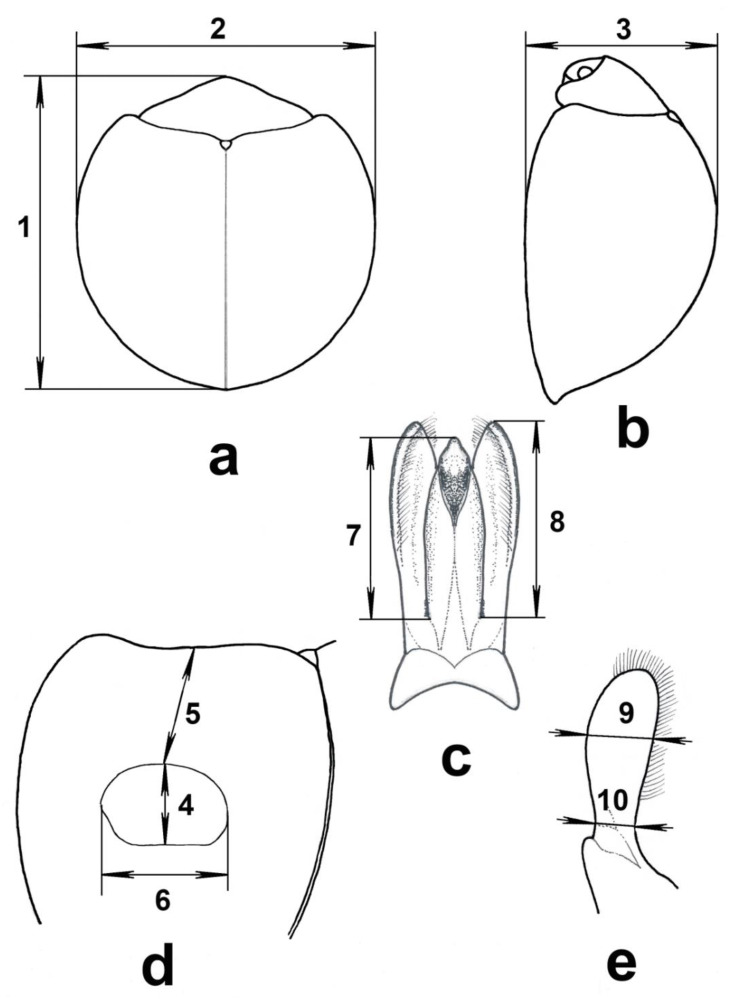
Examined morphological details. (**a**) General dorsal view; (**b**) general lateral view; (**c**) male penis guide and parameres, ventral view; (**d**) left elytron, dorsal view; (**e**) male paramere, lateral view. Measured characteristics: 1—body length, 2—body width, 3—body height, 4—length of the elytral marking along midline in the plane of the marking, 5—distance from the elytral marking to the base of elytron in the plane of elytron, 6—maximal width of the elytral marking in the plane of the marking, 7—length of penis guide from the junction with paramere to the apex, 8—length of paramere from the junction with the penis guide to the apex, 9—maximal width of paramere, 10—width of paramere at the constriction near the base.

Qualitative characteristics were studied by comparison with the “reference” samples, i.e., the specimens with the most clear manifestation of the character, as adopted by Bontems [46] and Bieńkowski and Orlova-Bienkowskaja [47]. The measurements are shown in Figure 1. Metric characteristics were studied under a stereomicroscope using a measuring eyepiece, and the division value was 0.07 mm for characters 1–3 and 0.02 mm for characters 4–10 (Figure 1).

### 2.3. Studied Characters

We studied 17 morphological characters, including all characters that were used by the authors of the original descriptions of species, revisions, and keys for this group of species to distinguish *Ch. kuwanae* and *Ch. renipustulatus* [7,33,36,37,43,45,46,47,48,49] as well as characters used for differentiation between *Ch. kuwanae* and other similar species:

1. Size of the elytral marking: maximal width of the elytral marking (Figure 1: 6)/body width (Figure 1: 2).
2. Body length (Figure 1: 1).
3. Proportion of the elytral marking: maximal width of the elytral marking (Figure 1: 6)/length of the elytral marking along the midline (Figure 1: 4).
4. Proportion of the body: body length (Figure 1: 1)/body width (Figure 1: 2).
5. Convexity of the body: body length (Figure 1: 1)/body height (Figure 1: 3).
6. Relative length of parameres: length of paramere (Figure 1: 8)/length of penis guide (Figure 1: 7).
7. Shape of parameres: maximal width of paramere (Figure 1: 9)/width of paramere at the constriction near the base (Figure 1: 10).
8. Location of the marking along the length of elytron (body length (Figure 1: 1)/distance from the elytral marking to base of elytron (Figure 1: 5)).
9. Marginated line of pronotum anteriorly: entire or narrowly interrupted at the middle (the interruption is not wider than half the width of the frons at the top between eyes) or broadly interrupted at middle.
10. Interspace between punctures on frons medially (smooth or obsoletely shagreen or distinctly shagreen).
11. Shagreened part on anterior lateral lobes of pronotum (absent or developed in a narrow region anteriorly or developed on the whole surface of the lobe).
12. Punctures of scutellum (large mixed with fine or fine only or absent).
13. Shape of scutellum (flat or weakly impressed or distinctly impressed).
14. Punctures at the elytral disk (fine, i.e., approximately 0.01 mm wide, or large, i.e., approximately 0.02 mm wide).
15. Shape of penis guide (with parallel sides in basal ½ or constricted basally and broadest in basal ¼).
16. Punctures on anterior lateral lobes of pronotum (fine, i.e., approximately 0.02 mm wide, or large, i.e., approximately 0.03 mm wide).
17. Punctures on frons (fine, i.e., approximately 0.01 mm wide, or large, i.e., approximately 0.02 mm wide).

The results of the study of morphological characteristics can be found in the Supplementary Material (Tables S1 and S2).

### 2.4. Criteria of Species and Subspecies

We adhere to the morphological concept of the species, i.e., we consider only morphologically different taxa. According to this concept, continuous variability in diagnostic characters should occur within the species, and different species should be distinguished by at least one diagnostic character without overlapping the limits of variability and with an unoccupied gap between them (hiatus) [50]. Exceptions to this rule are sibling species, different morphs within the species, and differences between males and females.

We use the morphological concept since this concept corresponds to the biological and ecological differences between populations [50] and because all known *Chilocorus* species exhibit morphological differences from each other. Though sibling species are known in Coleoptera [51], they are very rare. No sibling species of *Chilocorus* have been described in the literature. There are no external differences between males and females in the species under consideration, and there are also no different morphs. This gives us the opportunity to talk about morphological species in the present work.

For subspecies, we follow the classical rule by Amadon [52], which is used in current taxonomy [53]: 97% of specimens in one sample should be separable from 97% of specimens in the other sample to qualify these samples as representing different subspecies. Amadon has shown that this rule is fulfilled for a metric character if the following inequalities are true:

$$\{\begin{array}{c} \left|M_{1}-M_{2}\right|\geq 3.24\sigma_{1}+0.68\sigma_{2}\\ \left|M_{1}-M_{2}\right|\geq 3.24\sigma_{2}+0.68\sigma_{1} \end{array}$$

where $M_{1}$ is the mean value of the variable in the first population, $M_{2}$ is the mean value of the variable in the second population, $\sigma_{1}$ is the standard deviation in the first population, and $\sigma_{2}$ is the standard deviation in the second population [52]. Subspecies must be defined on diagnosability, not on mean differences [54]. We use the classical statistical method for the distinguishing of the subspecies because this is the only method appropriate for distinguishing of the subspecies currently used in zoology [54,55]. Unfortunately, the overwhelming majority of insect subspecies are currently described and revised without any statistical treatment.

## 3. Results

### 3.1. Size of Elytral Marking

According to Silvestri [7], the marking in *Ch. kuwanae* is smaller than that in *Ch. renipustulatus*. Savoyskaya [43] and Kuznetsov [36,37] noted that this marking is large in *Ch. renipustulatus* and very small in *Ch. kuwanae*. Kamiya [33] noted that this marking is very small in *Ch. kuwanae*.

Our measurement of the elytral marking in specimens from Europe and the Far East showed that the limits of the variation in this character in these two populations strongly overlap (Table 2). There is no hiatus between these populations; therefore, this character cannot differentiate them at the species level. In 72% of our specimens from Europe, the size of the elytral marking is within the range of variability of our specimens from the Far East, and in 78% of our specimens from the Far East, the size of the elytral marking is within the range of variability of our specimens from Europe. This character also cannot differentiate these populations at the subspecies level because it does not meet the subspecies criteria:

$$\left|M_{1}-M_{2}\right|=0.05\;3.24\sigma_{1}+0.68\sigma_{2}=0.13\;3.24\sigma_{2}+0.68\sigma_{1}=0.09$$

Therefore, $\left|M_{1}-M_{2}\right|<3.24\sigma_{1}+0.68\sigma_{2}$ and $\left|M_{1}-M_{2}\right|<3.24\sigma_{2}+0.68\sigma_{1}$.

### 3.2. Body Length

According to Kuznetsov [36], *Ch. renipustulatus* is 2.7–3.7 mm long, and *Ch. kuwanae* is 3.5–4.8 mm long, i.e., very little overlap of these limits of variability takes place. However, we did not find such a difference between the examined samples from Europe and the Far East. The ranges of variability of our samples strongly overlap: 3.41–5.18 mm in our specimens from Europe and 3.34–4.64 mm in our specimens from the Far East (Table 2). In 73% of our specimens from Europe, the body length is within the range of variability of our specimens from the Far East, and in 78% of our specimens from the Far East, the body length is within the range of variability of our specimens from Europe. The populations from Europe and the Far East do not correspond to Amadon’s criteria of subspecies by this character:

$$\left|M_{1}-M_{2}\right|=0.45\;3.24\sigma_{1}+0.68\sigma_{2}=1.20\;3.24\sigma_{2}+0.68\sigma_{1}=0.96$$

### 3.3. Proportion of the Elytral Marking

According to Silvestri [7], the elytral marking in *Ch. kuwanae* is less transverse than that in *Ch. renipustulatus*. Savoyskaya [43] and Kuznetsov [37] mentioned transverse and sometimes rounded markings in *Ch. kuwanae*, and transverse markings in *Ch. renipustulatus*.

The average proportion of elytral markings in our sample from Europe (1.52) is greater than that in our sample from the Far East (1.20). However, the ranges of variability strongly overlap (1.19–2.02 and 0.96–1.50). In 52% of our specimens from Europe, the proportion of elytral markings is within the range of variability of our specimens from the Far East, and in 45% of our specimens from the Far East, the proportion of elytral markings is within the range of variability of our specimens from Europe (Table 2). The populations from Europe and the Far East do not correspond to Amadon’s criteria of subspecies by this character:

$$\left|M_{1}-M_{2}\right|=0.32\;3.24\sigma_{1}+0.68\sigma_{2}=0.61\;3.24\sigma_{2}+0.68\sigma_{1}=0.38$$

### 3.4. Proportion of the Body

According to Silvestri [7], *Ch. kuwanae* is narrower than *Ch. renipustulatus*. However, in our samples, the ranges of variability are almost the same: 1.06–1.30 in Europe and 0.98–1.30 in the Far East (Table 2). The populations from Europe and the Far East do not correspond to Amadon’s criteria of subspecies:

$$\left|M_{1}-M_{2}\right|=0.01\;3.24\sigma_{1}+0.68\sigma_{2}=0.19\;3.24\sigma_{2}+0.68\sigma_{1}=0.17$$

### 3.5. Convexity of the Body

According to Silvestri [7], *Ch. kuwanae* is less convex than *Ch. renipustulatus*. We did not find differences in specific or subspecific levels in the samples from Europe and the Far East according to the convexity of the body. The ranges of variability of our samples are almost the same: 1.78–2.50 in Europe and 1.86–2.58 in the Far East (Table 2). The populations from Europe and the Far East do not correspond to Amadon’s criteria of subspecies:

$$\left|M_{1}-M_{2}\right|=0.06\;3.24\sigma_{1}+0.68\sigma_{2}=0.51\;3.24\sigma_{2}+0.68\sigma_{1}=0.34$$

### 3.6. Relative Length of Parameres

According to Savoyskaya [43], the paramere is distinctly longer than the penis guide in *Ch. kuwanae*, and the paramere is slightly longer than the penis guide in *Ch. renipustulatus*. According to Li et al. (2018), parameres are as long as 1.5× the penis guide length in *Ch. kuwanae*. We did not find differences in samples from different regions at the species or subspecies level (Table 2):

$$\left|M_{1}-M_{2}\right|=0.04\;3.24\sigma_{1}+0.68\sigma_{2}=0.16\;3.24\sigma_{2}+0.68\sigma_{1}=0.12$$

### 3.7. Shape of Parameres

According to Kamiya [33], a slightly clavated paramere is found in *Ch. kuwanae*. There are no species or subspecies differences in our samples from different regions (Table 2):

$$\left|M_{1}-M_{2}\right|=0.16\;3.24\sigma_{1}+0.68\sigma_{2}=0.66\;3.24\sigma_{2}+0.68\sigma_{1}=0.41$$

### 3.8. Location of the Marking along the Length of the Elytron

Gordon [49] used this character to distinguish *Ch. kuwanae* from similar American species. We did not find differences in samples from different regions at either the species level or the subspecies level according to this character (Table 2):

$$\left|M_{1}-M_{2}\right|=0.18\;3.24\sigma_{1}+0.68\sigma_{2}=0.93\;3.24\sigma_{2}+0.68\sigma_{1}=0.70$$

### 3.9. Marginated Line of Pronotum Anteriorly

According to Kamiya [33], the marginated line is interrupted in *Ch. kuwanae* and fully developed in the similar species *Chilocorus esakii* Kamiya, 1959. We did not find differences in the samples from different regions according to this character at either the species level or the subspecies level (Table 3). The majority of specimens from Europe as well as the majority of specimens from the Far East have broadly interrupted marginated lines.

### 3.10. Interspace between Punctures on Frons Medially

According to Kamiya [33], a distinctly developed microsculpture of the frons occurs in *Ch. kuwanae*, and the interspace between punctures is smooth in the similar species *Ch. esakii*.

In most specimens from Europe, the interspace between punctures is smooth (58 ± 4%), while in most specimens from the Far East, the interspace is distinctly shagreen (63 ± 5%). Intermediate states (frons are obsoletely shagreen) occur in specimens from the Far East (34 ± 5%) and Europe (36 ± 4%). There is no hiatus between them, since all three variants occur in both regions. The populations cannot be qualified as subspecies since they do not correspond to Amadon’s criteria.

### 3.11. Shagreened Part on Anterior Lateral Lobes of Pronotum

According to Kamiya [33], the shagreened part is absent in *Ch. kuwanae*. In our material, this character varies in Europe and the Far East, but the large shagreened part dominates in both regions. There are no species or subspecies differences (Table 3).

### 3.12. Punctures of Scutellum

According to Kamiya [33], punctures of the scutellum are fine in *Ch. kuwanae*. There are no species or subspecies differences in our samples (Table 3). The punctures of scutellum are fine in most specimens from Europe (71 ± 3%) and the Far East (51 ± 5%).

### 3.13. Shape of Scutellum

According to Kamiya [33], the scutellum in *Ch. kuwanae* is flat. In our material, an intermediate state (slightly impressed scutellum) is present in 45–55% of specimens from both regions. There are no species or subspecies differences (Table 3).

### 3.14. Punctures at Elytral Disk

According to Silvestri [7], *Ch. kuwanae* has more distinct puncturation of the body than *Ch. renipustulatus*. We did not find differences in the specific or subspecific levels of elytral puncturation in the samples from different regions (Table 3).

### 3.15. Shape of Penis Guide

According to Li et al. [45], the penis guide is constricted basally, and it is broadest in the basal ¼ in *Ch. kuwanae*; while it is parallel in the basal half in the similar species *Ch. esakii*. In our materials, the former shape of the penis guide prevails in all regions. There are no species or subspecies differences in our samples from different regions (Table 3).

### 3.16. Punctures on Anterior Lateral Lobes of Pronotum

According to Kamiya [33], the fine punctures on anterior lobes occur in *Ch. kuwanae*, and larger punctures are found in the similar species *Ch. esakii*. In our material, fine punctures prevail in all regions. There are no species or subspecies differences according to this character (Table 3).

### 3.17. Punctures on Frons

According to Kamiya [33], punctures on frons in *Ch. kuwanae* are larger than in *Ch. renipustulatus*. However, our study has shown that there is no hiatus between the populations from Europe and the Far East according to this character: both fine and large puncturation is common in both populations. Most European specimens (89%) have fine punctures, while most specimens from the Far East (65%) have large punctures (Table 3). However, this difference is not enough to qualify these populations as subspecies.

## 4. Discussion

**Figure 2:**
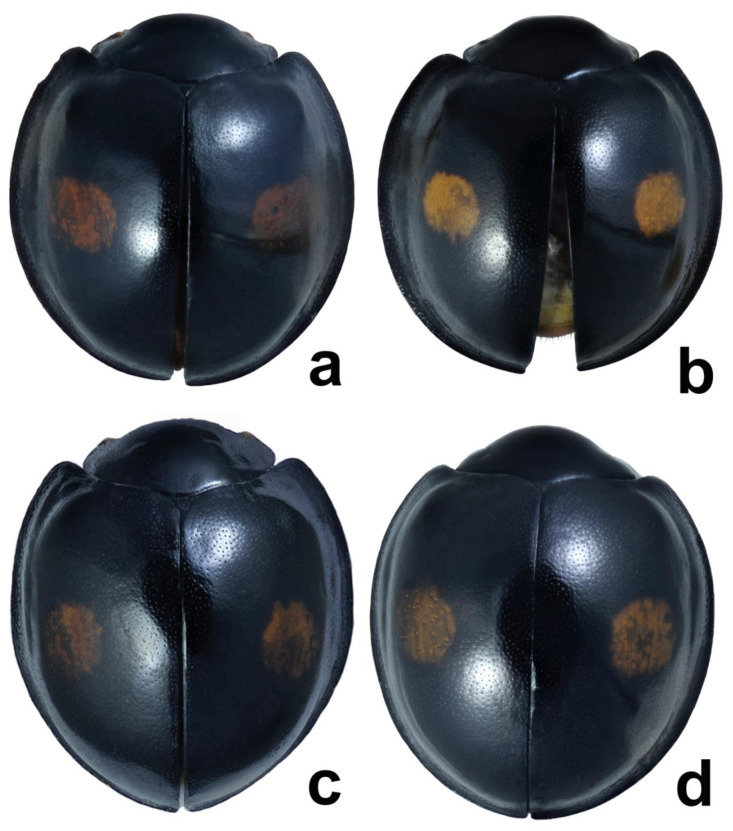
*Chilocorus renipustulatus*, total dorsal view. (**a**) Male from Germany; (**b**) male from Japan, Honshu; (**c**) female from Germany, Saxony; (**d**) female, Japan, Honshu.

**Figure 3:**
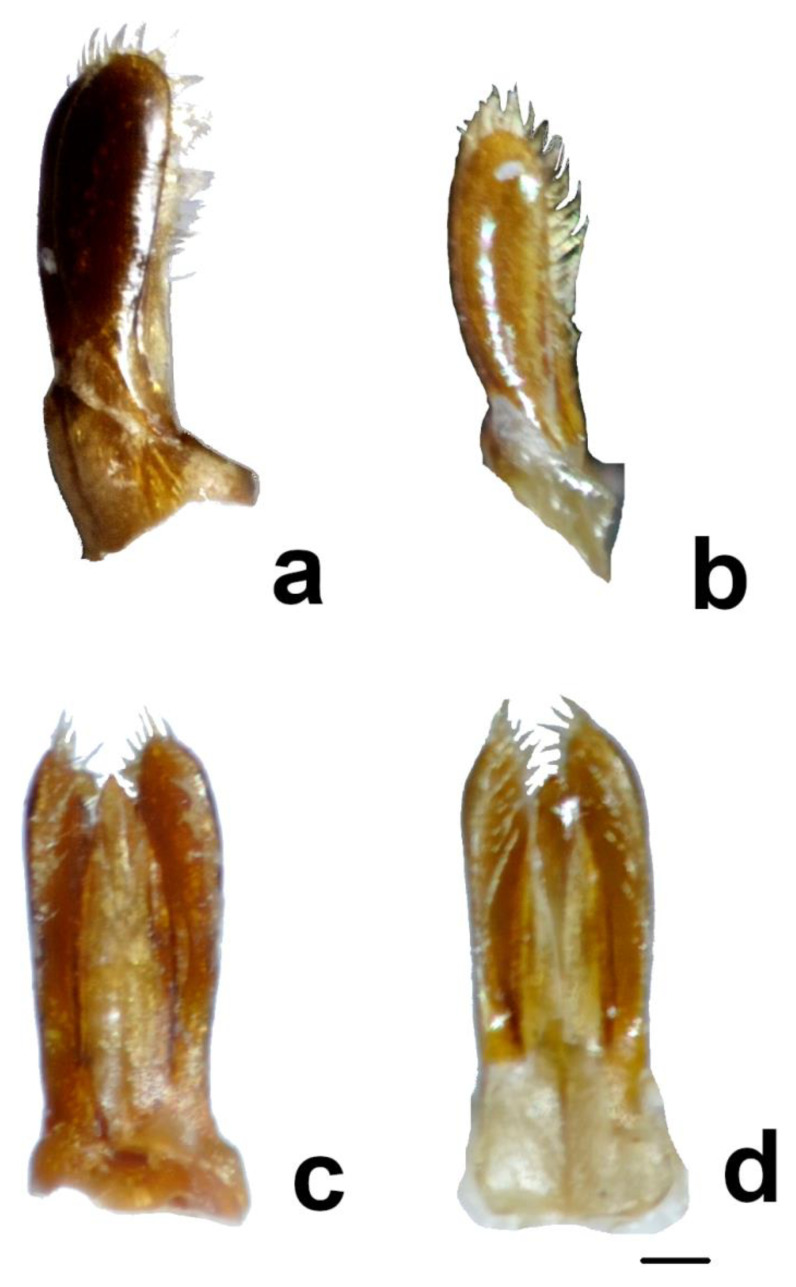
*Chilocorus renipustulatus*, male genitalia: parameres and penis guide. (**a**,**c**) Male from Germany; (**b**,**d**) male from Japan, Honshu; (**a**,**b**) lateral view, (**c**,**d**) ventral view. Scale bar: 0.1 mm.

Until now, the diagnostic differences between *Ch. renipustulatus* and *Ch. kuwanae* were considered by taxonomists without an analysis of the geographic and individual variability in morphological characters. We studied the variability of a number of morphological characteristics on the materials from different locations, including the type localities of both taxa, for the first time. As a result, no differences between *Ch. kuwanae* and *Ch. renipustulatus* were found, neither at the species level nor at the subspecies level. Therefore, we confirm the synonymy of *Ch. kuwanae* and *Ch. renipustulatus* established by Bieńkowski [34] (Figure 2 and Figure 3).

*Chilocorus kuwanae* and *Ch. renipustulatus* are two names for the same species, which means that the introductions of “*Ch. kuwanae*” from the Far East to Europe and the Caucasus were in fact introductions of specimens of *Ch. renipustulatus*. The plant protection experts believed that they had introduced the species outside its native range, while in fact, they introduced specimens from one part of the range of the species to another part of the range of the same species. Our study has shown that thorough taxonomical revision with the study of morphological variability should be conducted before the introduction of any species to a new region. The genus *Chilocorus* should be further investigated, including molecular studies.

## 5. Conclusions

(1) No differences between the specimens from Asia (Japan and Sakhalin) and Europe were found at specific or subspecific levels. *Chilocorus kuwanae* is a junior synonym of *Ch. renipustulatus*.
(2) The releases of “*Chilocorus kuwanae*” in Europe and the Caucasus did not represent classical biological control since the same species was native to these regions.
(3) A thorough taxonomical revision with the study of morphological variability should be conducted before the introduction of any species to new regions.
(4) Taxonomical conclusions based on morphological studies should be confirmed by statistical methods.

## Figures and Tables

**Table 1:** Material examined.

Region	Number of Specimens	Number of Males
Europe (Austria, Czech Republic, Hungary, Poland, Germany, Slovakia, European Russia)	174	58
Far East (Sakhalin, Japan)	107	38
Total number of specimens	281	96

**Table 2:** Metric characters of the populations from Europe and the Far East. M—mean value, σ—standard deviations, SE—standard error. Numbers of characters correspond to their numbers in the text. E from FE—ratio of specimens from Europe within the range of variability of the samples from the Far East, FE from E—ratio of specimens from the Far East within the range of variability of the samples from Europe.

		Europe			Far East				
№	Characters	$Min_{1}$	$Max_{1}$	$M_{1}\pm \;\mathrm{SE}$	$Min_{2}$	$Max_{2}$	$M_{2}\pm \;\mathrm{SE}$	E from FE, %	FE from E, %
1	Size of the elytral marking	0.26	0.44	0.34 ± 0.01	0.21	0.36	0.29 ± 0.01	72	78
2	Body length	3.41	5.18	4.43 ± 0.02	3.34	4.64	3.98 ± 0.03	73	99
3	Proportion of the elytral marking	1.19	2.02	1.52 ± 0.01	0.96	1.50	1.20 ± 0.01	52	45
4	Proportion of the body	1.06	1.30	1.17 ± 0.01	0.98	1.30	1.16 ± 0.01	99	95
5	Convexity of the body	1.78	2.50	2.15 ± 0.01	1.86	2.58	2.09 ± 0.01	99	99
6	Relative length of parameres	1.02	1.22	1.15 ± 0.01	1.07	1.29	1.18 ± 0.01	98	92
7	Shape of parameres	1.33	2.20	1.67 ± 0.02	1.25	1.86	1.51 ± 0.02	88	95
8	Location of the marking along the length of elytron	2.96	4.17	3.47 ± 0.02	2.71	4.05	3.28 ± 0.02	98	91

**Table 3:** Qualitative characters of the populations from Europe and the Far East. SE—standard error. Numbers of characters correspond to their numbers in the text.

№	Character		Europe Percentage ± SE, %	Far East Percentage ± SE, %
9	Marginated line of pronotum anteriorly	broadly interrupted	89 ± 2	76 ± 4
		narrowly interrupted	10 ± 2	24 ± 4
		entire	1 ± 1	0 ± 0
10	Interspace between punctures on frons medially	smooth	58 ± 4	4 ± 2
		obsoletely shagreen	36 ± 4	34 ± 5
		distinctly shagreen	6 ± 2	63 ± 5
11	Shagreened part on anterior lateral lobes of pronotum	absent	16 ± 3	5 ± 2
		developed in narrow region anteriorly	25 ± 3	14 ± 3
		developed on whole surface of lobe	60 ± 4	81 ± 4
12	Punctures of scutellum	absent	5 ± 1	1 ± 1
		fine only	71 ± 3	51 ± 5
		large mixed with fine	24 ± 3	48 ± 5
13	Shape of scutellum	flat	52 ± 4	24 ± 4
		weakly impressed	45 ± 4	55 ± 5
		distinctly impressed	3 ± 1	21 ± 4
14	Punctures at elytral disk	fine	3 ± 1	11 ± 3
		large	97 ± 1	89 ± 3
15	Shape of penis guide	parallel in basal half	7 ± 3	3 ± 3
		constricted basally	93 ± 3	97 ± 3
16	Punctures on anterior lateral lobes of pronotum	fine	83 ± 3	76 ± 4
		large	17 ± 3	24 ± 4
17	Punctures on frons	fine	89 ± 2	35 ± 5
		large	11 ± 2	65 ± 5

## Supplementary Materials

The following are available online at https://www.mdpi.com/2075-4450/11/6/368/s1, Table S1: Studied characters of the specimens from Europe, Table S2: Studied characters of the specimens from the Far East.
